# *In Situ* Crosslinked Hydrogel Depot for Sustained Antibody Release Improves Immune Checkpoint Blockade Cancer Immunotherapy

**DOI:** 10.3390/nano11020471

**Published:** 2021-02-12

**Authors:** Jihoon Kim, David M. Francis, Susan N. Thomas

**Affiliations:** 1 Parker H. Petit Institute for Bioengineering and Bioscience, Georgia Institute of Technology, 315 Ferst Dr NW, Atlanta, GA 30332, USA; jkim3441@gatech.edu (J.K.); dfrancis32@gatech.edu (D.M.F.); 2 George W. Woodruff School of Mechanical Engineering, Georgia Institute of Technology, 315 Ferst Dr NW, Atlanta, GA 30332, USA; 3 School of Chemical and Biomolecular Engineering, Georgia Institute of Technology, 315 Ferst Dr NW, Atlanta, GA 30332, USA; 4 Wallace H. Coulter Department of Biomedical Engineering, Georgia Institute of Technology, 313 Ferst Dr NW, Atlanta, GA 30332, USA and Emory University, 201 Dowman Drive, Atlanta, GA 30322, USA; 5 Winship Cancer Institute, Emory University School of Medicine, 1365-C Clifton Road NE, Atlanta, GA 30322, USA

**Keywords:** intradermal immunotherapy, immune checkpoint blockades, sustained release, *in situ* crosslinked hydrogel

## Abstract

The therapeutic inhibition of immune checkpoints, including cytotoxic T lymphocyte-associated protein (CTLA)-4 and programmed cell death 1 (PD-1), through the use of function blocking antibodies can confer improved clinical outcomes by invigorating CD8^+^ T cell-mediated anticancer immunity. However, low rates of patient responses and the high rate of immune-related adverse events remain significant challenges to broadening the benefit of this therapeutic class, termed immune checkpoint blockade (ICB). To overcome these significant limitations, controlled delivery and release strategies offer unique advantages relevant to this therapeutic class, which is typically administered systemically (e.g., intravenously), but more recently, has been shown to be highly efficacious using locoregional routes of administration. As such, in this paper, we describe an *in situ* crosslinked hydrogel for the sustained release of antibodies blocking CTLA-4 and PD-1 signaling from a locoregional injection proximal to the tumor site. This formulation results in efficient and durable anticancer effects with a reduced systemic toxicity compared to the bolus delivery of free antibody using an equivalent injection route. This formulation and strategy thus represent an approach for achieving the efficient and safe delivery of antibodies for ICB cancer immunotherapy.

## 1. Introduction

Immune checkpoint blockade (ICB) therapy has transformed medical oncology within the last decade [1,2]. Based on function blocking antibodies, this therapeutic class works by inhibiting the suppressive signaling activities of immune checkpoints, including cytotoxic T lymphocyte-associated protein (CTLA)-4 and programmed cell death (PD)-1, amongst other targets [1,2,3,4]. The most well-established are therapies inhibiting the functions of CTLA-4, which is expressed on T lymphocytes and outcompetes CD28 for engagement with CD80/CD86 on antigen presenting cells (APCs) to suppress cytotoxic CD8^+^ T cell priming [1,2,3]. In addition, the receptor-ligand pair that is now the most widely targeted is PD-1 expressed by T cells, which engages with ligands expressed by APCs to attenuate CD8^+^ T cell activation or cancer cells, resulting in the evasion of tumor immune surveillance [1,2,4]. Accordingly, the administration of antibodies blocking CTLA-4 or aPD-1 signaling (aCTLA-4 and aPD-1) enables CD8^+^ T cell-mediated anticancer immunity to be invigorated [1,2,3,4]. However, response rates for ICB therapy are generally low and treatment is often associated with adverse immune-related events [1,2,3,4]. Improving the therapeutic effects and minimizing off-target toxicities are key to decreasing cancer mortality in the next decade [1,2,3,4,5].

We recently reported the benefit conferred with respect to the therapeutic efficacy and safety profile by both intratumoral (i.t.) administration and intradermal (i.d.) injection to the tissue ipsilateral (i.l.) to the tumor compared to the conventional systemic administration most often used in preclinical studies and the standard practice used clinically [5]. The locoregional i.t. or i.l. administration of aCTLA-4 and aPD-1 antibodies in combination was revealed to not only facilitate an enhanced systemic response of proliferating CD8^+^ T cells, but also reduce the systemic toxicity associated with low antibody doses accumulating within off-target systemic and non-tumor-associated tissues. Promisingly, administration into the tissues i.l. to the tumor site, which only resulted in antibody accumulation within lymph nodes co-draining the tumor, was as effective as i.t. therapy. Moreover, this administration scheme conferred dose sparing benefits, suggesting the potential for ICB antibodies administered into peripheral tissues rather than systemically. This also presents multiple advantages over direct i.t. injection, considering the difficulty of the direct administration into tumors within deep tissues [5,6,7], with such sites potentially providing insufficient volumes for injection [5,7], and the frequent absence of tertiary lymphoid structures/niches within the tumor, which are thought to play a pivotal role in facilitating CD8^+^ T cell infiltration, survival, and instruction [8,9,10]. Numerous therapeutic antibodies, including Humira^®^, Herceptin^TM^, and Xgeva^®^, are administered into the subcutaneous or other tissue space in a free or depot-forming formulation [11,12,13]. In this way, these sustained or triggered release formulations allow antibody effects to be exerted over sustained periods of time [13,14]. Additionally, circulating levels of subcutaneously administered antibody have been shown to be proportional to the injected dose [15]. However, the potential for the continuous delivery of a low dose ICB antibody from an injection into the periphery, but outside the tumor site, to lead to improved therapeutic outcomes compared to one high dose, has not been established.

Drug delivery systems (DDSs) offer numerous advantages for improving the drug biodistribution and pharmacokinetic/pharmacodynamic profiles, thus providing a high potential to improve the therapeutic efficacy and ameliorate the side effects of therapeutics [16,17,18]. However, despite the explosive growth of ICB-related research, the development of DDSs for ICB has only recently begun. To date, the ICB therapy field has mostly focused on the potential for ICB immunotherapy in diverse tumor types [1,2,3,4,5] or the effects in combination with other existing therapeutics with or without formulations with various DDSs [19,20]. Few DDSs for ICB have been reported, and those that have are primarily designed to result in antibody release or accumulation within the tumor directly after i.t. administration of ICB antibody-containing nanoparticles [21], hydrogels [22,23,24], scaffolds [25,26,27], and microneedles [28,29] or an i.v. injection of ICB antibody-loaded nanoformulations [30,31,32]. However, the potential for sustained release formulations to enhance the effects of ICB therapy administered extratumorally using locoregional administration was recently demonstrated by our group [5]. Specifically, we leveraged thermosensitive hydrogels formed from bare Pluronic^®^ F127 to prolong the ICB antibody half-life at the site of injection ~20 fold (from ~2 to 20 h) [5]. Further prolonging this *in vivo* residence time has the potential to further improve not only the therapeutic benefits, but also patient compliance, by minimizing the need for repeated administrations.

**Scheme 1:**
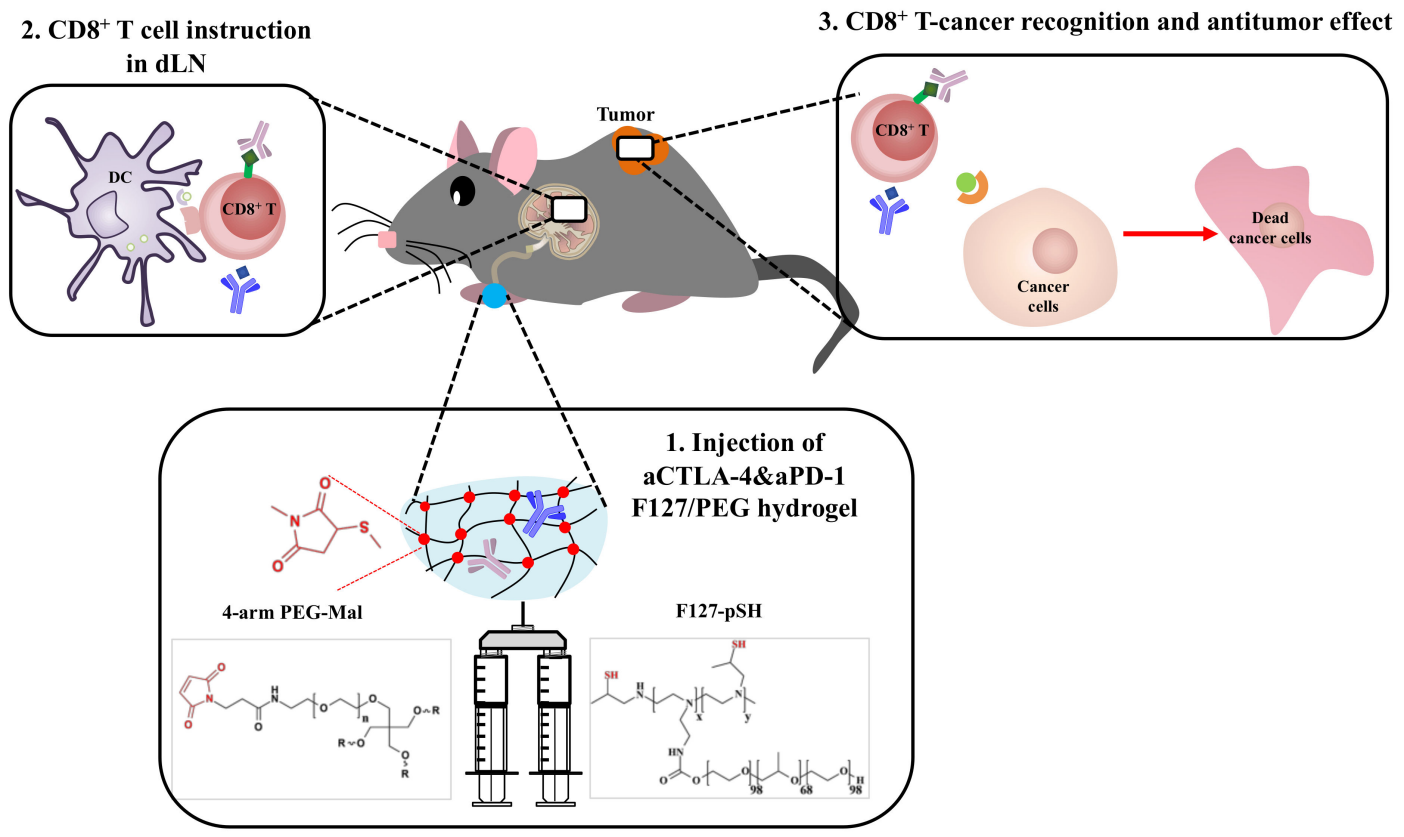
Schematic of the immune checkpoint blockade (ICB) antibody releasing hydrogel for antitumor therapy. ICB antibodies are released from intradermally injected hydrogels in a sustained manner. As a result, the benefits of ICB for T cell instruction are prolonged, improving the anti-tumor effects of ICB immunotherapy.

Herein, we demonstrate the therapeutic potential of the sustained release of ICB antibodies from *in situ* crosslinked hydrogels injected i.l. in terms of anticancer effects (Scheme 1). In detail, thermosensitive and biocompatible Pluronic^®^ F127 was chemically conjugated to small molecular weight (MW) branched polyethyleneimine (BPEI) [33], followed by the thiolation of polymer amine groups [34]. The resultant F127-pSH dissolved in aCTLA-4 and aPD-1 antibody containing aqueous solvent was mixed with 4-arm poly(ethylene glycol) functionalized with maleimide (4-arm PEG-Mal), resulting in an *in situ* crosslinked hydrogel. We hypothesized that this formulation would result in a long lasting, crosslinked F127/PEG hydrogel that would release ICB antibodies in a sustained manner. When injected within the skin, more durable and efficient anticancer effects were observed with insignificant systemic toxicity.

## 2. Materials and Methods

### 2.1. Materials

Pluronic^®^ F127, tris(2-carboxyethyl)phosphine, 4-nitrophenyl chloroformate (*p*-NPC), and Red Blood Cell Lysing Buffer Hybri-Max^TM^ were provided by Sigma Aldrich (St. Louis, MO, USA). Propylene sulfide was purchased from TCI America (Portland, OR, USA). Phosphate-buffered saline (PBS) with or without calcium and magnesium, dichloromethane, methyl alcohol, ethyl ether, deuterium oxide (D_2_O, Cambridge Isotope Laboratories, Andover, MA, USA), Amicon^®^ ultra-15 centrifugal tube (molecular weight cut-off (MWCO) 10 kDa, Millipore, Bellerica, MA, USA), Spectra/Por 7 standard regenerated cellulose dialysis membrane (MWCO 10kDa, Spectrum Industries, Los Angeles, CA, USA), an alanine aminotransferase (ALT) activity colorimetry/fluorometry assay kit (Biovision, Mountain View, CA, USA), and an aspartate aminotransferase (AST) activity colorimetric assay kit (Biovision, CA, USA) were obtained from VWR Scientific (USA). Alexa Fluor^TM^ 647 NHS Ester (Invitrogen™, Carlsbad, CA, USA) was provided by Thermo Fisher Scientific (USA). 4-arm PEG-Mal (molecular weight (MW) 20KDa) and BPEI (MW 600 Da) were purchased from JenKem Technology USA (Allen, TX, USA) and BeanTown Chemical (Hudson, NH, USA), respectively. aCTLA-4 (clone 9H10) and aPD-1 (clone RMP1-14) were purchased from BioXCell (Lebanon, NH, USA).

### 2.2. Synthesis and Characterization of F127-pSH

F127-pSH was synthesized via a three-step process. Pluronic^®^ F127 (10 g) in dichloromethane (70 mL) was activated with *p*-NPC (1.6 g) for two days, followed by precipitation under cold ethyl ether. BPEI600 Da (5 g) in dichloromethane (50 mL) was reacted with the activated F127 for two days and dialyzed with a dialysis membrane (MWCO 10 kDa) against deionized water (D.W.) until a yellow byproduct was not detected, followed by freezing drying. The thermosensitive sol-gel transition behaviors of resultant F127-BPEI were investigated using a vial tilting method. The *in vitro* stability of F127 and F127-BPEI was evaluated by weighing the hydrogels incubated in DMEM containing 10% FBS in a 37 °C water bath. F127-pSH was synthesized by reacting F127-BPEI (1.6 g) in methanol (70 mL) with propylene sulfide (5 mL) at 60 °C for two days and precipitated under cold ethyl ether three times. The composition of resultant F127-BPEI and F127-pSH in D_2_O was confirmed by ^1^H nuclear magnetic resonance spectroscopy (^1^H NMR) with Bruker Advance 400 MHz FT-NMR.

### 2.3. Preparation of F127/PEG Hydrogel

Prior to being used for the formation of hydrogel, F127-pSH (100 mg) in PBS without calcium and magnesium (2 mL) was reacted with tris(2-carboxyethyl)phosphine (50 mg) for 30 min to reduce disulfide bonds and then purified five times by centrifugal dialysis with an Amicon^®^ ultra-15 centrifugal tube (MWCO 10 kDa). Thiol contents of the reduced F127-pSH were quantified by Ellman’s assay. The results were recorded as the mean ± standard deviation (SD) (*n* = 4), with statistical analysis being conducted by one-way ANOVA supported by Prism 9 (GraphPad Software, San Diego, CA).

Additionally, 2.7% (*w*/*v*) F127/PEG hydrogel was formed by mixing 4% (*w*/*v*) reduced F127-pSH (8 μL in PBS without calcium and magnesium), antibodies containing PBS (16 μL in PBS with calcium and magnesium), and 8% (*w*/*v*) 4-arm PEG-Maleimide (MW 20 kDa) (6 μL in PBS without calcium and magnesium) at a molar ratio of 1:1 thiol to maleimide.

### 2.4. Characterization of F127/PEG Hydrogel

The rheological properties of the hydrogel were investigated by dynamic oscillatory strain and frequency sweeps on a Discovery HR-2 rheometer (TA Instruments) with an 8 mm diameter and flat geometry at an angular frequency (ω) of 1–10 rad s^−1^ (Plate SST 8 mm Smart-Swap, TA Instruments). The results were recorded as the mean ± SD (*n* = 3–4), with statistical analysis being conducted by one-way ANOVA supported by Prism 9 (**** *p* < 0.0001, *** *p* < 0.001, ** *p* < 0.01, and * *p* < 0.05). Scanning electron microscopy (SEM) images were obtained from lyophilized hydrogel by using Hitachi SU-8230 at an accelerating voltage of 1 kV and 10 μA emission current. The *in vitro* stability of hydrogels was evaluated by weighing the hydrogel incubated in DMEM containing 10% FBS in a 37 °C water bath. Alexa Fluor™ 647-labeled aCTLA-4 was prepared for the *in vitro* and *in vivo* drug release test. In brief, 20 µL of 10 mM Alexa Fluor^TM^ 647 NHS Ester in DMSO was mixed with 1.8 mg of aCTLA-4 in 200 µL PBS at room temperature for 2 h. A Sepharose^®^ CL-6B column (GE Healthcare) and five centrifugal dialysis processes with an Amicon^®^ ultra-15 centrifugal tube (MWCO 10 kDa) enabled the purification of Alexa Fluor™ 647-labeled aCTLA-4. aCTLA-4 release from hydrogel was observed in Alexa Fluor™ 647-labeled aCTLA-4 containing 90 μL 2.7% (*w*/*v*) hydrogel incubated in 180 μL PBS in a 37 °C water bath. At a pre-determined time point, supernatant was collected and 180 μL fresh PBS was added. The fluorescence of supernatants was recorded for the aCTLA-4 release test. The results were recorded as the mean ± SD (*n* = 4), with statistical analysis being conducted by two-way ANOVA supported by Prism 9.

### 2.5. In Vitro Biocompatibility of F127/PEG Hydrogel

NIH3T3 was seeded onto 96-well plates at a density of 1 × 10^4^ cells/well and then incubated overnight. Polymer solutions were prepared by reacting F127-pSH and 4-arm PEG-mal to produce a final concentration of 1 mg/mL in 10% FBS containing DMEM. The polymer solutions with serial dilution were added to the seeded cells and incubated for 2 d. In order to investigate the cytotoxicity of the leach-out byproduct of the hydrogel, 100 μL hydrogel was formed in 96-well plates and 100 μL 10% FBS containing DMEM was added, followed by 2 d incubation. The supernatants were transferred to the NIH3T3-seeded 96-well plates and incubated for 2 d. An Alamarblue assay was conducted to evaluate the cytotoxicity of the polymer and leach-out byproducts. The fluorescence signal from cells incubated with 10% FBS DMEM lacking polymers was used to represent the 100% cell viability. The results were recorded as the mean ± SD (*n* = 5–6), with statistical analysis being conducted by two-way ANOVA supported by Prism 9.

### 2.6. In Vivo Stability of F127/PEG Hydrogel and aCTLA-4 Release Test from the Hydrogel

All animal studies were conducted in accordance with Georgia Tech’s Institutional Animal Care and Use Committee (IACUC) and performed in the Physiological Research Laboratory (PRL) at the Georgia Institute of Technology. The *in vivo* stability of the F127/PEG hydrogel was investigated by measuring the size of 30 μL of 2.7% (*w*/*v*) hydrogel intradermally injected into dorsal skin of Balb/C mice. *In vivo* aCTLA-4 release was evaluated by measuring the fluorescence where 30 μL of 2.7% (*w*/*v*) hydrogel containing Alexa Fluor™ 647-labeled aCTLA-4 was injected and the amount of remaining fluorescence over time quantified with an IVIS^®^ Spectrum instrument (Perkin Elmer, Waltham, MA, USA). The results were recorded as the mean ± standard error of the mean (SEM), with statistical analysis being conducted by two-way ANOVA supported by Prism 9.

### 2.7. In Vivo Anticancer Therapy with aCTLA-4 and aPD-1 Releasing F127/PEG Hydrogel

IACUC approved all animal experiments that were performed in the PRL at the Georgia Institute of Technology. A murine breast tumor model was established by inoculating 3 × 10^5^ 4T1 cells intradermally in the left mammary fatpad of 6–12 week old Balb/C mice on day 0. In total, 30 μL of saline, free aCTLA-4+aPD-1, and aCTLA-4+aPD-1 containing 2.7% (*w*/*v*) F127/PEG hydrogel (dose equivalent to 30 or 50 μg of each aCTLA-4 and aPD-1) was administered to the skin ipsilateral to the tumor (i.l.) on day 10. Blood serum was collected 2 d after treatment, which was further analyzed using an ALT and AST assay kit to investigate treatment effects on liver toxicity. The tumor volume was calculated as the cuboidal volume based on tumor measurements in three orthogonal dimensions. The experiments were performed twice, with four mice in each group. The results were recorded as the mean ± SEM, with statistical analysis being conducted by two-way ANOVA supported by Prism 9.

### 2.8. Statistics

*In vitro* and *in vivo* results are presented as the mean ± SD and mean ± SEM, respectively. Formulation and/or treatment effects were analyzed by one-way ANOVA and two-way ANOVA with Tukey post-hoc using Prism 9. *P* values are given as **** *p* < 0.0001, *** *p* < 0.001, ** *p* < 0.01, and * *p* < 0.05.

## 3. Results and Discussion

Pluronic^®^ F127 was selected as a polymeric backbone for the ICB antibody-releasing hydrogel depot due to its amphiphilic nature, which facilitates the sustained release of various hydrophobic and hydrophilic drugs [35]. However, the thermosensitive hydrogel made by Pluronic^®^ F127 itself has a short residence time *in vivo*, as well as in aqueous solution (Supplementary Materials Figure S1). Accordingly, there have been numerous reported hydrogels developed by crosslinking Pluronic^®^ backbones *in situ*. Among the various crosslinking strategies, including the Michael-type addition reaction, Click chemistry, the Diels–Alder reaction, the ultraviolet-mediated thiol-ene reaction, the enzyme-mediated reaction, host–guest interactions, and divalent cation-mediated coordination [36], the thiol-maleimide crosslinking method was employed because it is free from the use of additional substrates, enzymes, cytotoxic ions, and light, which can affect the structure or effectiveness of ICB antibodies.

Small molecular BPEI was conjugated to F127 via a *p*-NPC chemistry to convert two low reactive hydroxyl groups in Pluronic^®^ F127 into highly reactive multi-arm amines (Supplementary Materials Figure S2). The successful conjugation of BPEI in F127-BPEI was demonstrated by ^1^H NMR by confirming the amine peaks in 2.6-2.9 ppm (Supplementary Materials Figure S3). F127-BPEI had a higher stability than F127, as confirmed by sol-gel transition curves (Supplementary Materials Figure S4 and Supplementary Materials Table S1), which can be attributed to the stabilization effects of hydrophilic BPEI in micelle–micelle interactions, as reported previously [33]. However, it was only able to retain its structure for one day (Supplementary Materials Figure S1), which justified the need for *in situ* crosslinked hydrogels for sustained ICB release *in vivo*. The secondary and primary amines of F127-BPEI were further converted into thiol groups to afford the F127-pSH by reacting them with propylene sulfide (Supplementary Materials Figure S2), as confirmed by ^1^H NMR showing methyl and tertiary proton peaks at 1.2–1.4 and 2.8–3.2 ppm, respectively (Supplementary Materials Figure S3) [34]. Ellman’s assay demonstrated that one F127-pSH contains 4.00 ± 0.04 thiol groups, calculated from the results that one F127-BPEI contains 0.82 ± 0.01 BPEI and 1 mg of resultant F127-pSH contains 305.6 ± 3.3 nmole thiol groups (Supplementary Materials Figure S5).

**Figure 1:**
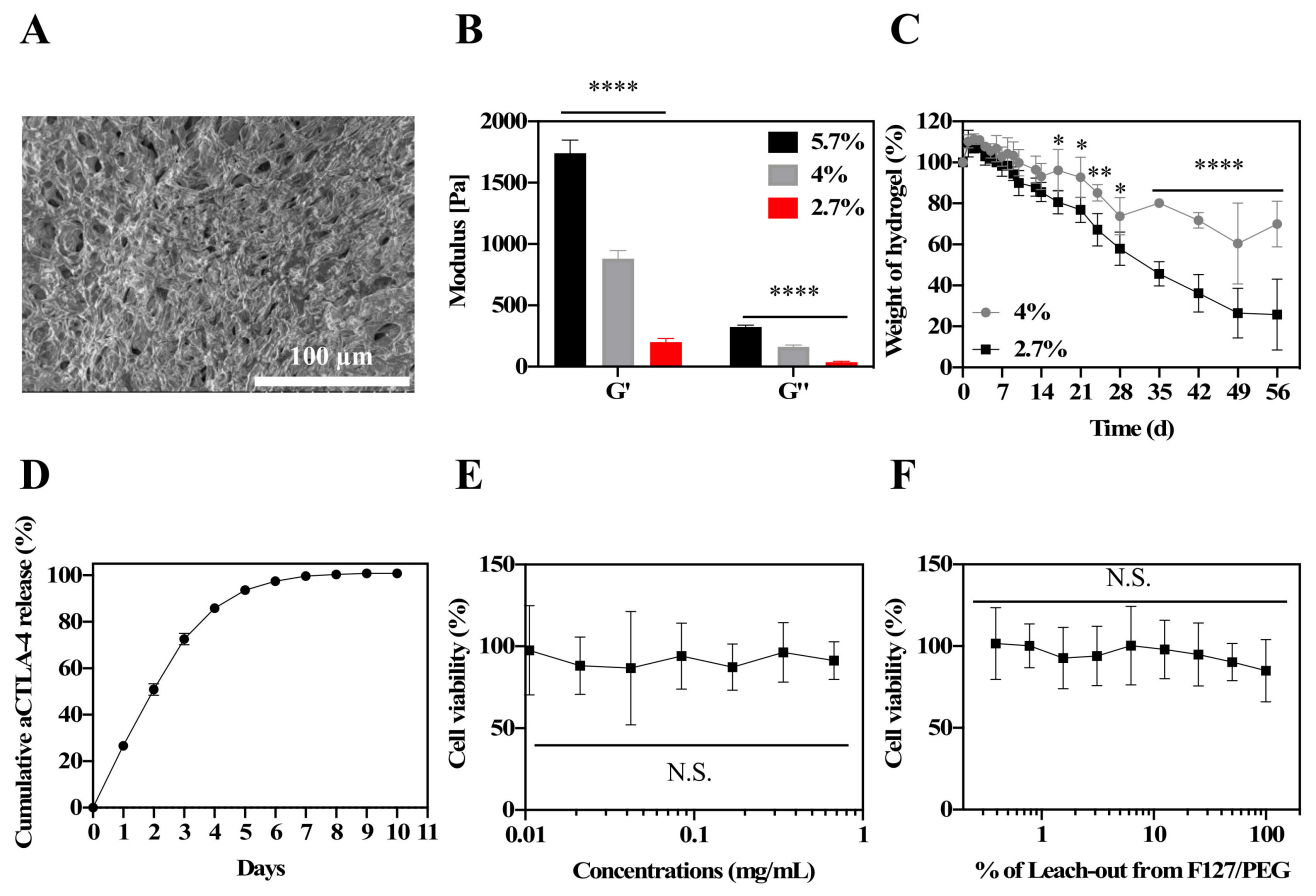
Characterization of *in situ* crosslinked F127/PEG hydrogel. (**A**) SEM images of F127/PEG hydrogel. (**B**) Rheology of F127/PEG hydrogels. (**C**) *In vitro* stability of F127/PEG hydrogels. (**D**) *In vitro* antibody (Alexa Fluor™ 647-labeled aCTLA-4) release from 2.7% (*w*/*v*) F127/PEG hydrogels. (**E**) Cytotoxicity of F127/PEG polymers on NIH3T3 cells. (**F**) Cytotoxicity of F127/PEG hydrogel leach-out extracts on NIH3T3 cells. **** p < 0.0001, *** p < 0.001, ** p < 0.01, and * p < 0.05, which were analyzed with a one-way ANOVA test for (**B**), (**E**), and (**F**), and a two-way ANOVA test for (**C**).

4-arm PEG-Mal was employed to form *in situ* crosslinked hydrogel because it is biocompatible, commercially available, and highly reactive with the thiol groups of F127-pSH. The *in situ* crosslinked hydrogel (F127/PEG) was formed by mixing 4% (*w*/*v*) F127-pSH solution and 8% (*w*/*v*) PEG-Mal solution at equivalent ratios of thiol and maleimide groups, diluted with saline to the final hydrogel concentration desired. The F127/PEG hydrogel was formed in 10 s (total hydrogel volume = 30 μL in 2.7% (*w*/*v*) hydrogel) to 1 min (total hydrogel volume = 1 mL in 2.7% (*w*/*v*) hydrogel) after mixing the solution, which exhibited porous structures, as revealed in scanning electron microscopy (SEM) images (Figure 1A). As expected, hydrogel with higher polymer concentrations resulted in higher measured storage (G’) and loss moduli (G’’) by rheology (Figure 1B, Supplementary Materials Figure S6). The 4% (*w*/*v*) polymer weight hydrogels exhibited notable resistances to spontaneous degradation compared to hydrogels formed from 2.7% (*w*/*v*) polymer weights, implying concentration-dependent modulation of the hydrogel stability (Figure 1C). As high polymer concentration solutions can be challenging to handle, and for forming homogenous hydrogels, the 2.7% (*w*/*v)* hydrogel was selected for further *in vitro* and *in vivo* studies. *In vitro*, the F127/PEG hydrogel exhibited sustained release of the aCTLA-4 antibody (*t*_1/2_ = 2.0 ± 0.1 d, Figure 1D). Suggestive of the favorable biocompatibility of the F127/PEG hydrogel, neither the polymer components comprising the F127/PEG hydrogel up to 1 mg/mL (Figure 1E) nor the leach-out extract from hydrogel (Figure 1F) exhibited cytotoxic effects against the mouse fibroblast cell line (NIH3T3) with 48 h co-incubation.

**Figure 2:**
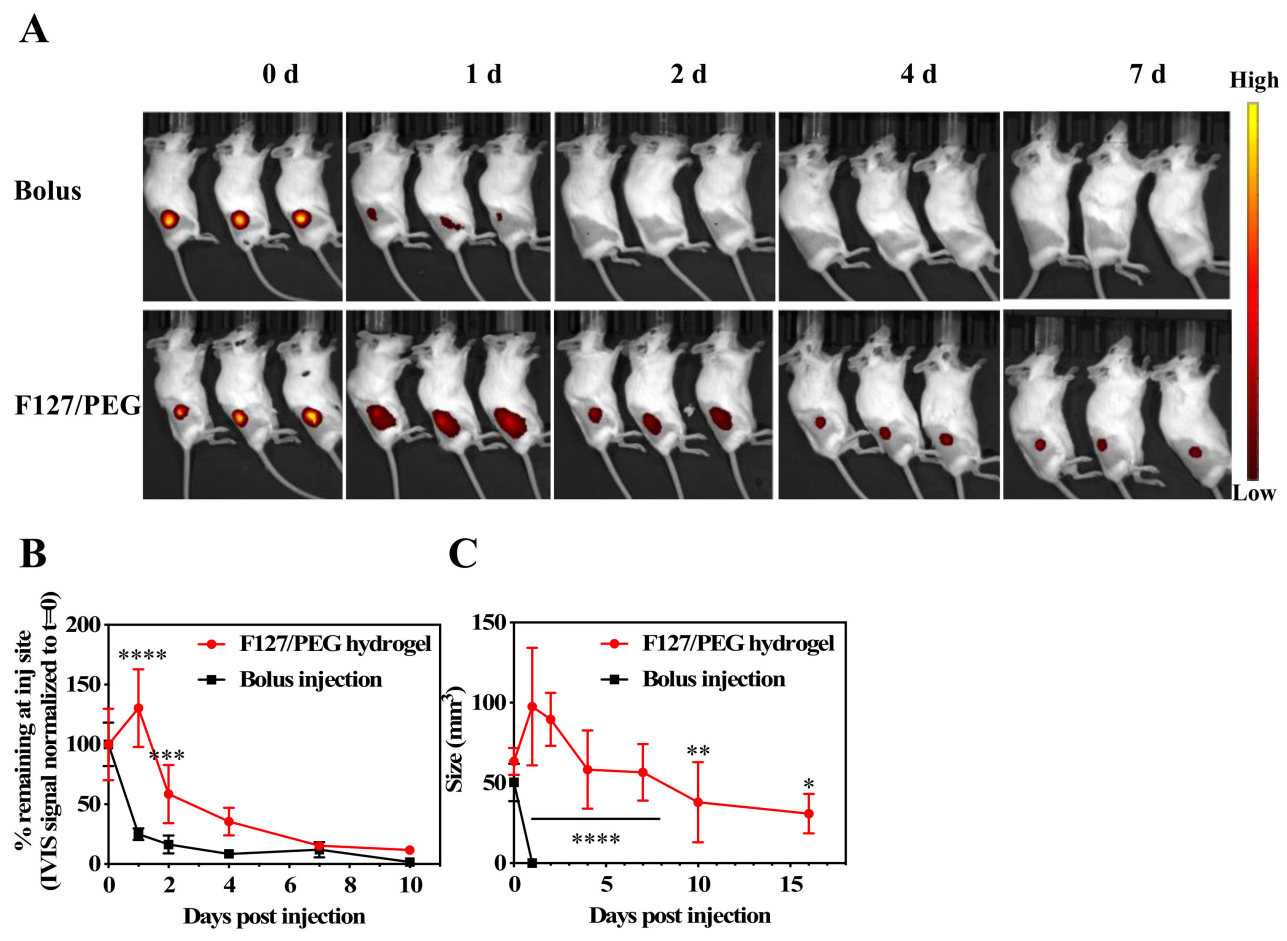
Antibody release from *in situ* crosslinked F127/PEG hydrogels *in vivo*. (**A**) Time-resolved IVIS images of Alexa Fluor™ 647-labeled aCTLA-4 antibody dermally injected with bolus or within 2.7% (*w*/*v*) F127/PEG hydrogels. (**B**) Quantification of IVIS fluorescence at the injection site. (**C**) *In vivo* stability of F127/PEG hydrogels. **** p < 0.0001, *** p < 0.001, ** p < 0.01, and * p < 0.05, which were analyzed with a two-way ANOVA test.

Whether F127/PEG hydrogels facilitate the sustained release of ICB antibodies *in vivo* was then investigated. Alexa Fluor^TM^ 647-labelled aCTLA-4 antibody in its free form or incorporated into F127/PEG hydrogels was injected into the dorsal skin of healthy Balb/C mice and the fluorescence signal was measured over seven days on an IVIS^®^ Spectrum (Perkin Elmer, MA, USA) (Figure 2A). A high fluorescent signal was found to only be sustained in animals in which the fluorescent antibody was delivered with F127/PEG hydrogels. While less than half of the aCTLA-4 antibody (25.0 ± 2.2%) injected as a bolus remained after one day, the antibody signal within F127/PEG hydrogels was detectable over the entire seven days and exhibited a significantly longer half-life (3.3 ± 0.4 d) (Figure 2B). Considering that dense hydrogel structures reduce the diffusion of water and drugs [28], sustained drug release is generally dependent on the stability of hydrogel. Indeed, the F127/PEG hydrogel retained its size for more than two weeks, although its size slowly reduced over time (Figure 2C). Therefore, the significantly prolonged release of ICB from F127/PEG hydrogels compared to bolus delivery can be ascribed to the long residence time of F127/PEG hydrogel *in vivo*.

**Figure 3:**
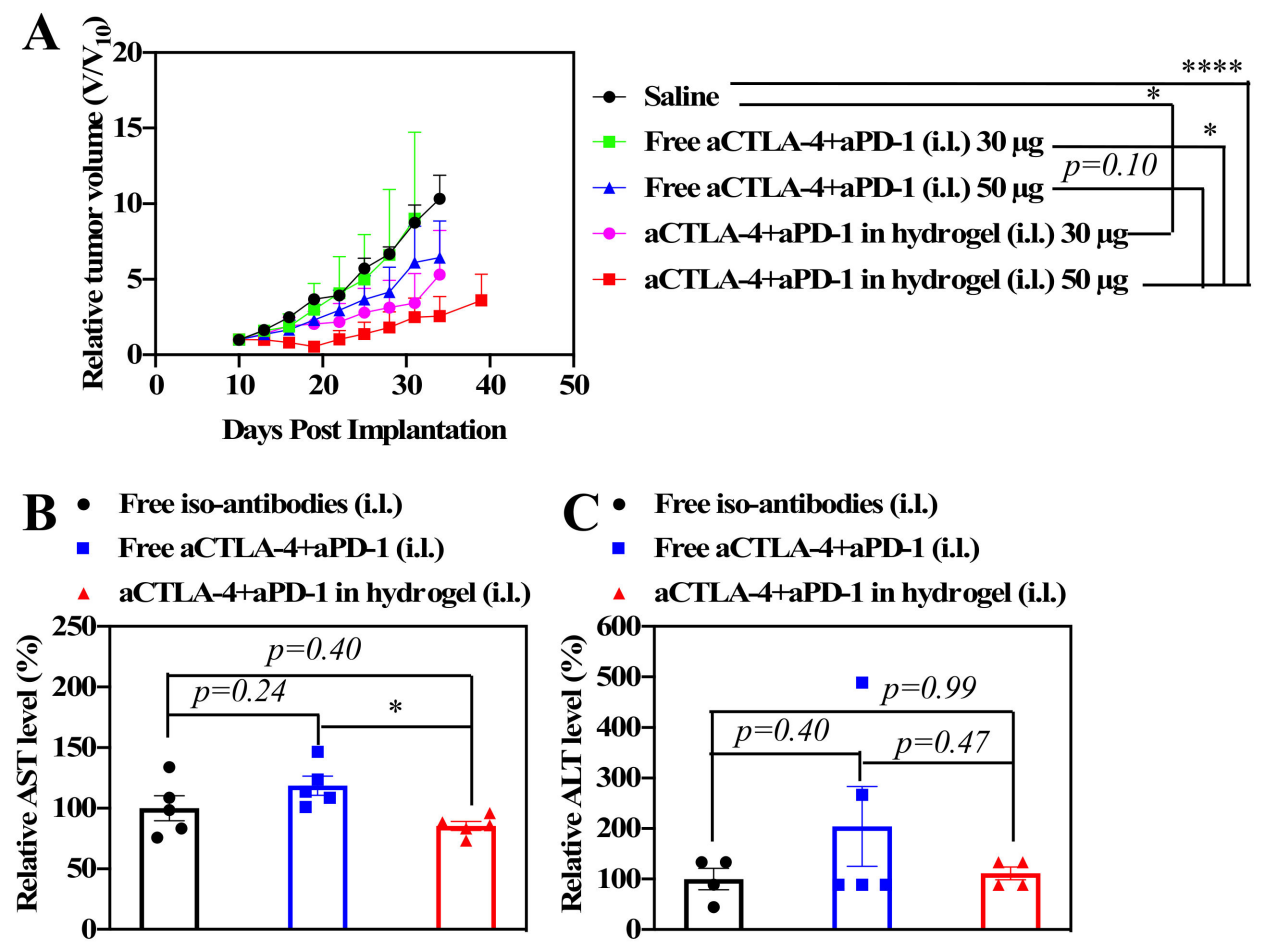
Antitumor effects of aCTLA-4+aPD-1 containing F127/PEG hydrogels *in vivo*. (**A**) Tumor size after one administration of saline, free aCTLA-4+aPD-1 (30 or 50 μg total dose of each monoclonal antibody (mAb) clone), or aCTLA-4+aPD-1 (30 or 50 μg total dose of each mAb clone) containing F127/PEG hydrogels injected intradermally (i.d.) into the tissue ipsilateral (i.l.) to the tumor on day 10. (**B**) Aspartate aminotransferase (AST) and (**C**) alanine aminotransferase (ALT) in blood 2 d after treatment of 50 μg aCTLA-4+aPD-1. (**A**) was analyzed with two-way ANOVA. (**B**) and (**C**) were analyzed with one-way ANOVA. **** p < 0.0001 and * p < 0.05.

In order to investigate the effects of the sustained release of ICBs in anticancer therapy, 4T1 murine breast tumor cells were inoculated into Balb/C mice on day 0 as a model of metastatic triple negative breast cancer. Following the administration of 30 μL of saline, free aCTLA-4+aPD-1, or aCTLA-4+aPD-1 containing F127/PEG hydrogels (dose equivalent to 30 or 50 μg of each aCTLA-4 and aPD-1) i.d. into the tissues i.l. to the tumor on day 10, the tumor size was measured to investigate the dose-dependent benefit of the F127/PEG hydrogel for ICB cancer immunotherapy. Overall, tumor growth was suppressed more effectively by 50 μg compared to 30 μg ICB from both bolus and hydrogel delivery, whereas the HG formulation improved the effects of aCTLA-4+aPD-1 compared to control groups for both ICB doses (Figure 3A). In addition, the aCTLA-4+aPD-1 antibody containing F127/PEG hydrogels had negligible effects on the systemic liver toxicity, whereas treatment with the free aCTLA-4+aPD-1 antibody resulted in slightly elevated serum alanine and aspartate aminotransferase levels (ALT and AST, respectively), which are indicative of liver toxicity (Figure 3B,C). The improved therapeutic efficacy and negligible systemic toxicity resulting from treatment with the aCTLA-4+aPD-1 antibody containing F127/PEG hydrogels compared to control groups may therefore be attributed to both the prolonged bioavailability of ICB antibodies in draining lymph node (dLN )and reduced levels within systemic tissues afforded by the hydrogel formulation [5]. Therefore, the overall results demonstrated that the sustained release of ICB antibodies by the crosslinked hydrogel leads to improved therapeutic outcomes with a negligible systemic toxicity.

In summary, thermosensitive and biocompatible Pluronic^®^ F127 is chemically conjugated to small molecular weight (MW) branched polyethyleneimine (BPEI), followed by the thiolation of amine groups. The resultant F127-pSH containing aCTLA-4 and aPD-1 antibodies is mixed with 4-arm PEG-Mal to afford *in situ* crosslinked hydrogel. The long-lasting hydrogel (F127/PEG) prolongs ICB antibody release to afford superior tumor control with simultaneous reductions in systemic toxicity.

## Supplementary Materials

The following are available online at https://www.mdpi.com/2079-4991/11/2/471/s1, Figure S1: Stability of F127 and F127-BPEI hydrogel in DMEM containing 10% FBS at 37 °C, Figure S2: Schematic of F127-pSH synthesis method, Figure S3: ^1^H NMR of F127, F127-BPE, and F127-pSH in D_2_O, Figure S4: Temperature- and concentration-dependent sol-gel transition of F127 and F127-BPEI, Figure S5: Quantification of weight density of polymer thiol groups in F127, F127-BPEI, and F127-pSH, Figure S6: Modulus of F127/PEG hydrogel at varying angular frequencies, Table S1: Sol-gel and gel-sol transition temperature of F127 and F127-BPEI.
