# Supplementary material for: In Situ Crosslinked Hydrogel Depot for Sustained Antibody Release Improves Immune Checkpoint Blockade Cancer Immunotherapy
Source: Nanomaterials (Basel). 2021 Feb 12;11(2):471. doi: 10.3390/nano11020471 (PMC7918828; doi:10.3390/nano11020471)
Supplement: Supplementary file 1 [file nanomaterials-11-00471-s001.pdf]

Supplementary Materials

Hydrogel Depot for Sustained Antibody Release Improves Immune Checkpoint Blockade Cancer Immunotherapy

*Jihoon Kim, David M. Francis and Susan N. Thomas**

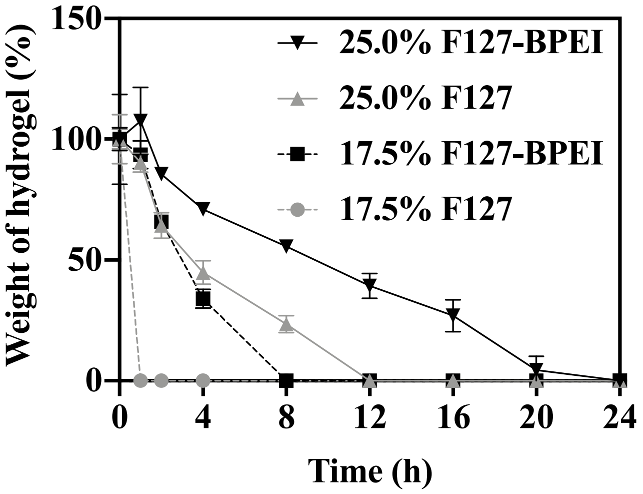

Supplementary Figure S1. Stability of F127 and F127-BPEI hydrogel in DMEM containing 10% FBS at 37 ˚C.

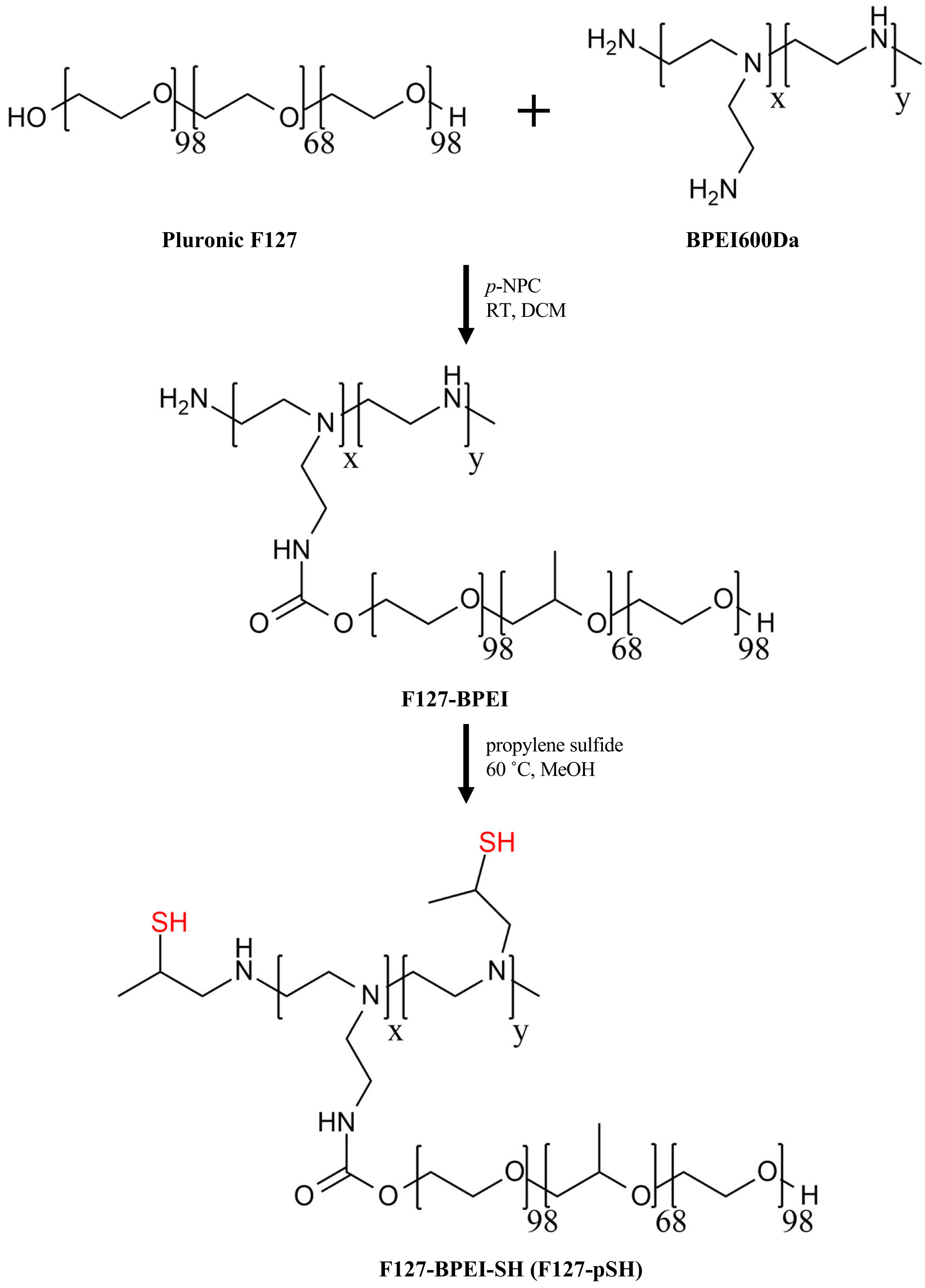

Supplementary Figure S2. Schematic of F127-pSH synthesis method.

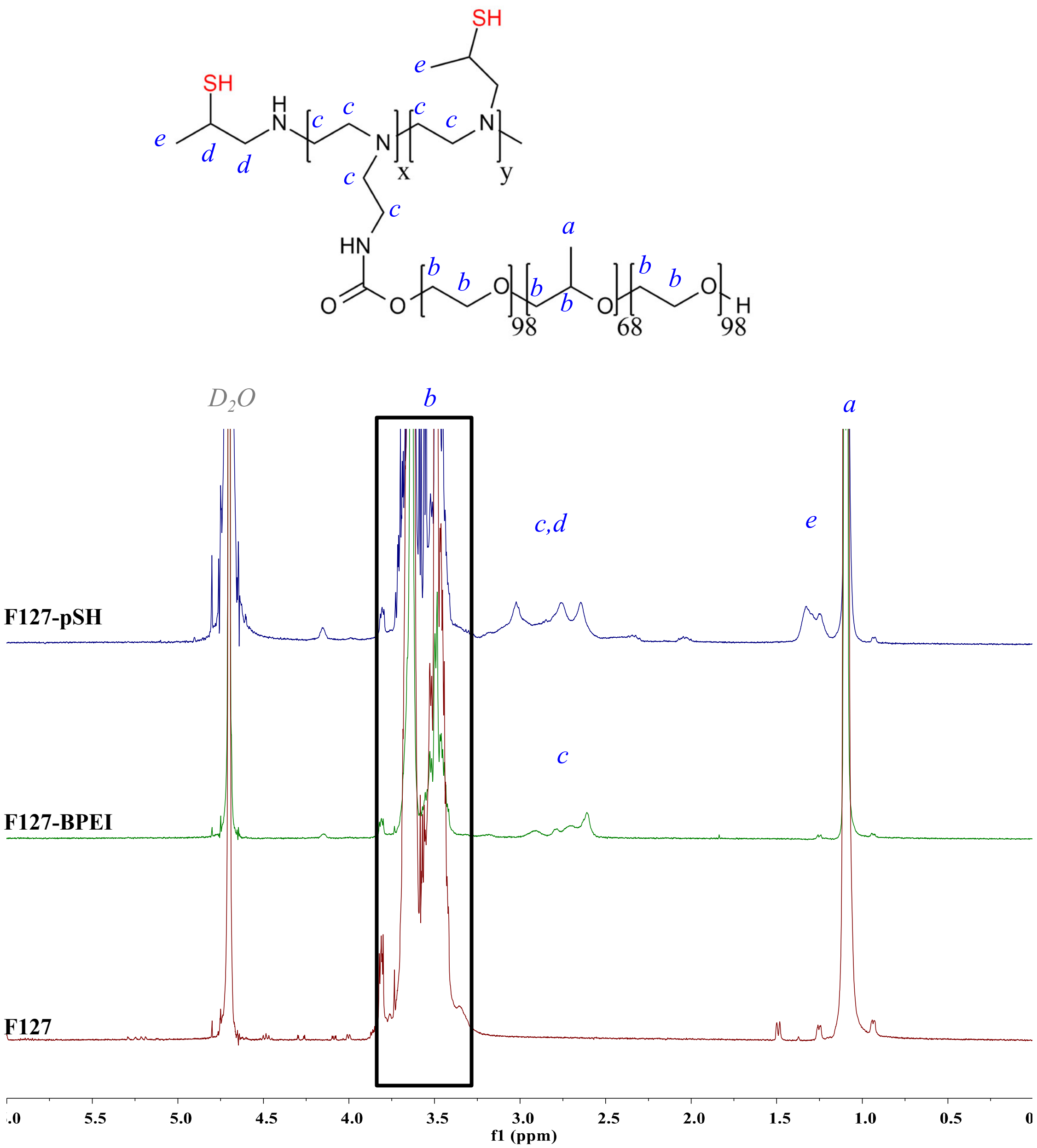

Supplementary Figure S3. ^1H NMR of F127, F127-BPE, and F127-pSH in D_2O.

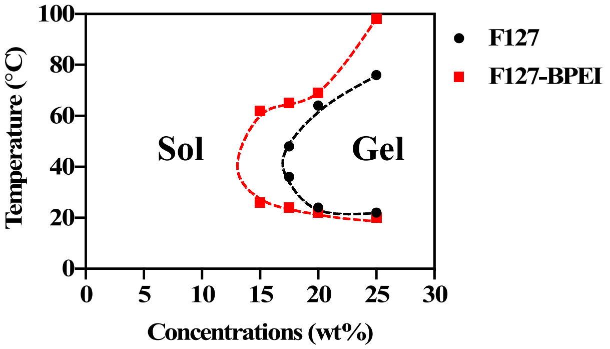

Supplementary Figure S4. Temperature- and concentration-dependent sol-gel transition of F127 and F127-BPEI.

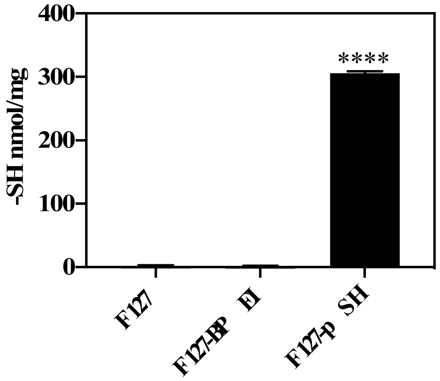

Supplementary Figure S5. Quantification of weight density of polymer thiol groups in F127, F127-BPEI, and F127-pSH.

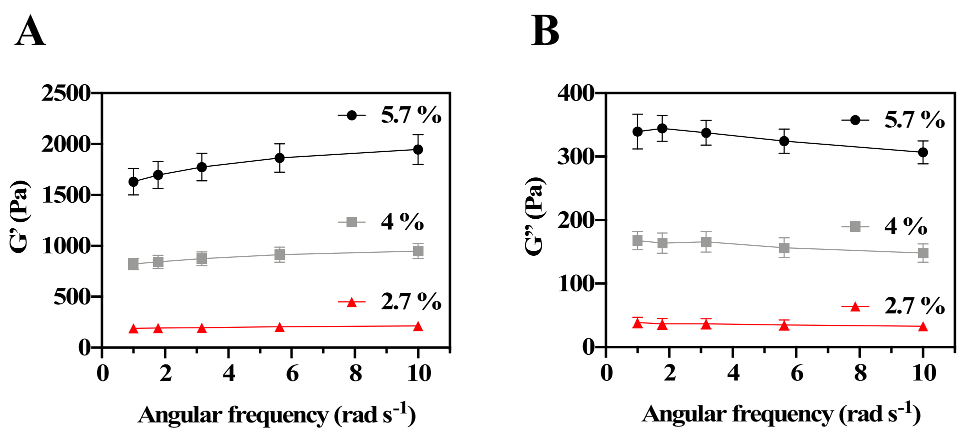

Supplementary Figure S6. Modulus of F127/PEG hydrogel at varying angular frequencies. (A) Storage modulus (G') of F127/PEG hydrogel at anguelar frequency 1-10 rad s^{-1}. (B) Loss modulus (G") of F127/PEG hydrogel at angular frequencies of 1-10 rad s^{-1}.

Supplementary Table S1. Sol-gel and gel-sol transition temperature of F127 and F127-BPEI.

% (w/v)	**Sol→gel transition temperature / Gel→seperation transition temperature**	
	**F127**	**F127-BPEI**
**1**	Not available (N.A.)	N.A.
**5**	N.A.	N.A.
**10**	N.A.	N.A.
**15**	N.A.	26 ˚C / 62 ˚C
**17.5**	36 ˚C / 48 ˚C	24 ˚C / 65 ˚C
**20**	24 ˚C / 64 ˚C	22 ˚C / 69 ˚C
**25**	22 ˚C / 76 ˚C	20 ˚C / 98 ˚C
